# An In vitro Caco2‐Based Model for Measuring Intestinal Bioadhesion Comparable to Ex vivo Models

**DOI:** 10.1002/smsc.202400461

**Published:** 2024-12-03

**Authors:** Eliyahu Drori, Valeria Rahamim, Dhaval Patel, Yamm Anker, Sivan Meir, Gal Uzan, Shira Somech, Chen Drori, Tal Tzadok, Aharon Azagury

**Affiliations:** ^1^ Department of Chemical Engineering and Biotechnology Ariel University Kiryat Hamada 3 Ariel 40700 Israel; ^2^ Department of Medical and Health Sciences Tel Aviv University Klachkin 35 Tel Aviv‐Yafo 6997801 Israel; ^3^ Department of Maurice and Gabriela Goldschleger School of Dental Medicine Tel Aviv University Klausner 6 Tel Aviv‐Yafo 6997801 Israel

**Keywords:** alginate, bioadhesion, Caco‐2 cells, chitosan, ex vivo in vitro models, gelatin, oral drug delivery systems

## Abstract

This study presents an in vitro model using Caco‐2 cells that can mimic the bioadhesion properties of the human intestinal epithelium, aiming to reduce the use of animal tissues, in line with the 3Rs principle—replacement, reduction, and refinement. Specifically, a texture analyzer was used to assess the bioadhesive strength of hydrogels (i.e., alginate (Alg), chitosan (Chit), and gelatin (Gel)) under various applied forces (20–200 mN) and contact times (120–420 s). The results demonstrate that the in vitro model effectively predicts the bioadhesive strength of the tested hydrogels to ex vivo tissues (i.e., from mice, sheep, and pigs), including the effects of applied force and contact time. Also provided is an analysis of the effect of microvilli morphology on bioadhesion where an inverse relationship was observed between microvilli linear density and bioadhesion strength, explaining the variability in results across animal models. This Caco‐2‐based model offers a practical, accessible, and cost‐effective alternative to current ex vivo methods used for measuring bioadhesion fracture strength. It can be integrated into standardized testing protocols, providing a more ethical and scientifically robust approach to advancing bioadhesive drug delivery system research.

## Graphical abstract

This study introduces an in vitro Caco‐2 cell model for measuring intestinal bioadhesion, offering an alternative to animal‐based ex vivo methods. Using a texture analyzer, the model predicts the bioadhesive properties of materials under varying forces and durations, replicating ex vivo findings. Moreover, the study provides valuable insights into the influence of microvilli morphology on bioadhesion.

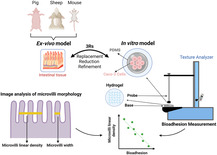

## 1 Introduction

Bioadhesive drug delivery systems (DDS) are mainly known for prolonging retention time at mucosal sites and improving therapeutic efficacy.^[^ ^1^, ^2^ ^]^ Bioadhesive DDS were primarily applied to the intestinal mucosa, where, for example, extended drug residence time enhanced its systemic absorption post‐oral administration in mice from 6% to 67%.^[^ ^3^ ^]^ Also, bioadhesive chitosan sponges significantly increased the bioavailability of medications, such as insulin, compared to their non‐adhesive counterparts.^[^ ^3^, ^4^ ^]^ Moreover, bioadhesives may also avoid first‐pass metabolism by binding to mucin proteins, offering a significant advantage in pharmacotherapy and localizing the intestine.^[^ ^5^ ^]^ Thus, assessing the bioadhesion property of materials is highly important in characterizing and analyzing bioadhesive materials.^[^ ^6^ ^]^ There are several types of bioadhesion tests,^[^ ^7^, ^8^ ^]^ most of which require using excised animal (e.g., rodent, pig) tissue.^[^ ^9^, ^10^ ^]^

However, ethical concerns (e.g., the 3 Rs principle—replacement, reduction, and refinement), availability issues, and logistical challenges associated with animal usage have spurred the search for alternative methodologies.^[^ ^11^ ^]^ Moreover, this search was further intensified when, in December 2022, the United States Food and Drug Administration (FDA) was instructed to repeal the requirement to test new drugs on animals before human testing and search and promote alternative in vitro models.^[^ ^12^ ^]^ Therefore, the main goal of this research was to develop an in vitro model to measure bioadhesion that is equivalent to using ex vivo tissues. To this end, we cultured Caco‐2 cells—the standard cell line of the human intestinal epithelium^[^ ^13^ ^]^—on polydimethylsiloxane (SYLGARD 184) designed to assess bioadhesive forces in a reproducible and ethical manner quantitatively. Caco‐2 cells were chosen because they assemble into microvilli morphology, which is similar to the morphology of the human small intestine, and form a monolayer of polarized cells with tight junctions.^[^ ^14^, ^15^ ^]^

To test our in vitro model, we used it to measure the bioadhesion of the standard biomaterials varying degrees of bioadhesion: Alg, Chit, and Gel.^[^ ^16^, ^17^ ^]^ These measurements were compared to ex vivo intestinal tissues from mice, pigs, and sheep. To this end, we used a texture analyzer at the applied forces of 20, 100, and 200 mN for different contact durations of 120, 270, and 420 s, measuring the strength required for detaching the two surfaces. The applied forces and contact times were selected to simulate the conditions in the intestinal tract during peristaltic movement. Specifically, the contact forces were chosen based on a study measuring peristaltic forces inside the intestines of a lamb, using an encapsulated prototype with a force sensor. This study found that peristaltic forces in the small intestine ranged between 0 and 180 mN.^[^ ^18^ ^]^ As for contact time, it was previously shown that bioadhesion does not significantly change beyond 7 min.^[^ ^10^ ^]^ In determining the contact times, we aimed to align with biological parameters relevant to gastrointestinal transit. For humans, small bowel transit ranges from 2 to 6 h, and colonic transit can extend from 10 to 59 h.^[^ ^19^ ^]^ With an average intestinal length of ≈795.5 ± 129 cm,^[^ ^19^ ^]^ peristaltic velocity ranges from 0.2 to 1.11 cm min^−1^, resulting in an average contact time of 0.45 to 2.5 min for our sample width of 0.5 cm.^[^ ^20^ ^]^ Similarly, for rodents—frequently employed as human models in oral drug delivery studies—residence time for non‐bioadhesive materials is ≈300 min,^[^ ^21^ ^]^ with an intestinal length of 55.5 cm and peristaltic velocity of 0.185 cm min^−1^, yielding an average contact time of about 2.7 min.^[^ ^22^ ^]^ These calculated times support our selected contact time, intended to mirror physiological residence time in the GI tract.

There needs to be a standardized animal model for bioadhesion studies, which is evident in the diversity of animal tissues employed in various research efforts. For instance, some studies have utilized pig intestinal tissues due to their anatomical and physiological similarities to human intestines.^[^ ^23^ ^]^ In contrast, others have chosen mouse models for their genetic and mucosal similarities.^[^ ^24^ ^]^ Additionally, sheep tissues have also been explored, and it was found that despite the differences in the digestive system from humans, the small intestine's structure is similar.^[^ ^25^ ^]^ While previous studies employed Caco‐2 cells mainly for permeability and cellular uptake assessments,^[^ ^26^, ^27^ ^]^ our study expands this approach by systematically evaluating bioadhesion fracture strength, which enables a novel comparison across species. Bioadhesive research has traditionally relied on ex vivo models for measuring bioadhesion fracture strength. To offer a practical, ethically aligned alternative, this study developed a Caco‐2‐based in vitro model designed for integration into standardized testing protocols. By mimicking the intestinal environment, this model provides a reliable and accessible method for advancing bioadhesive drug delivery research while reducing the need for animal tissues.

This study examines the influence of intestinal microvilli morphology on bioadhesion. Histologically, Caco‐2 cells and human, mouse, pig, and sheep intestinal tissues were analyzed to measure the density and width of the microvilli. This dual approach can enhance understanding of how intestinal morphology impacts bioadhesion interactions and thus can provide a more comprehensive evaluation of these properties than the in vitro model. While ex vivo animal tissues are often employed, their notable anatomical and physiological differences from human tissues can sometimes result in less reliable outcomes, suggesting improved models are needed.^[^ ^28^ ^]^ The mucus thickness and composition differences between animal and human tissues may affect bioadhesion studies.

## 2 Results and Discussion

The bioadhesion fracture strengths of Alg, Chit, and Gel were measured against the small intestines of mice, pigs, sheep, and our Caco‐2‐based in vitro model using a texture analyzer. As shown previously, three factors affect bioadhesion: the contact time, applied force, and, of course, the specific materials and tissues used.^[^ ^10^ ^]^ Thus, we evaluated the prediction ability of our in vitro model by assessing the effects of contact time and applied forces for three common bioadhesives: Alg, Gel, and Chit.

### 2.1 Comparative Analysis of Contact Time Effect on Bioadhesion

**Figure 1:**
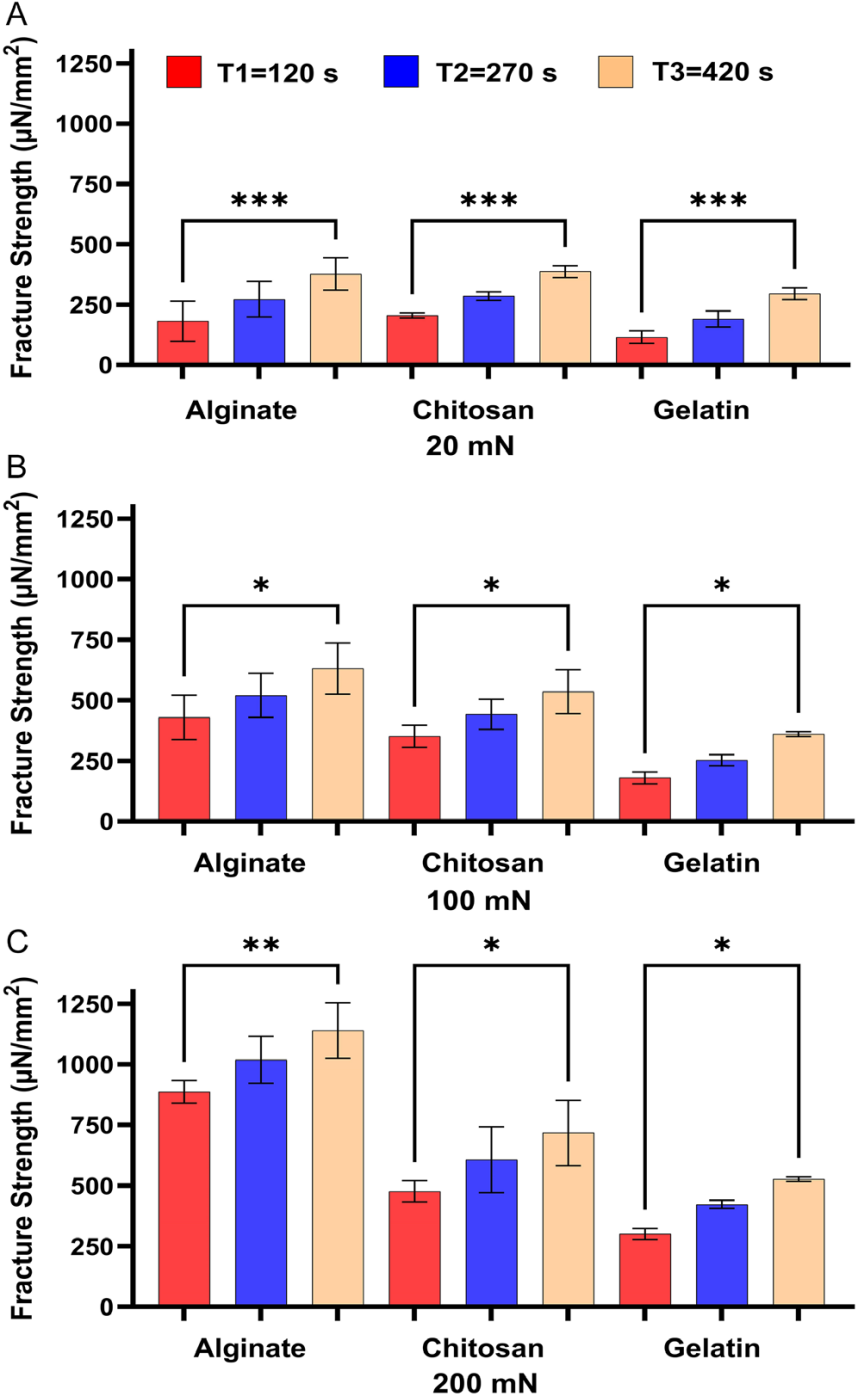
The effect of contact time on the bioadhesion of Alg, Chit, and Gel to the small intestines of mice at the applied forces of A) 20 mN, B) 100 mN, and C) 200 mN. One‐way ANOVA was applied to determine statistical significance. Values represent the mean ± SD of *n* = 4; *p* values denoted as *p < 0.05, **p < 0.01, and ***p < 0.001.

**Figure 2:**
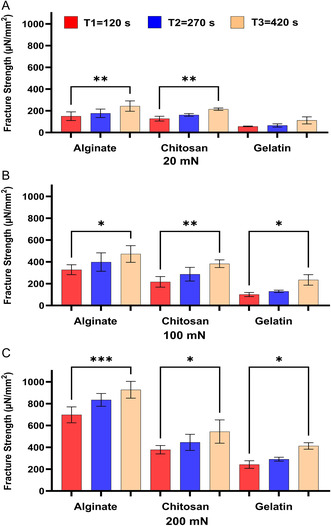
The effect of contact time on the bioadhesion of Alg, Chit, and Gel to the small intestines of pigs at the applied forces of A) 20 mN, B) 100 mN, and C) 200 mN. One‐way ANOVA was applied to determine statistical significance. Values represent the mean ± SD of *n* = 4; *p* values denoted as *p < 0.05, **p < 0.01, and ***p < 0.001.

**Figure 3:**
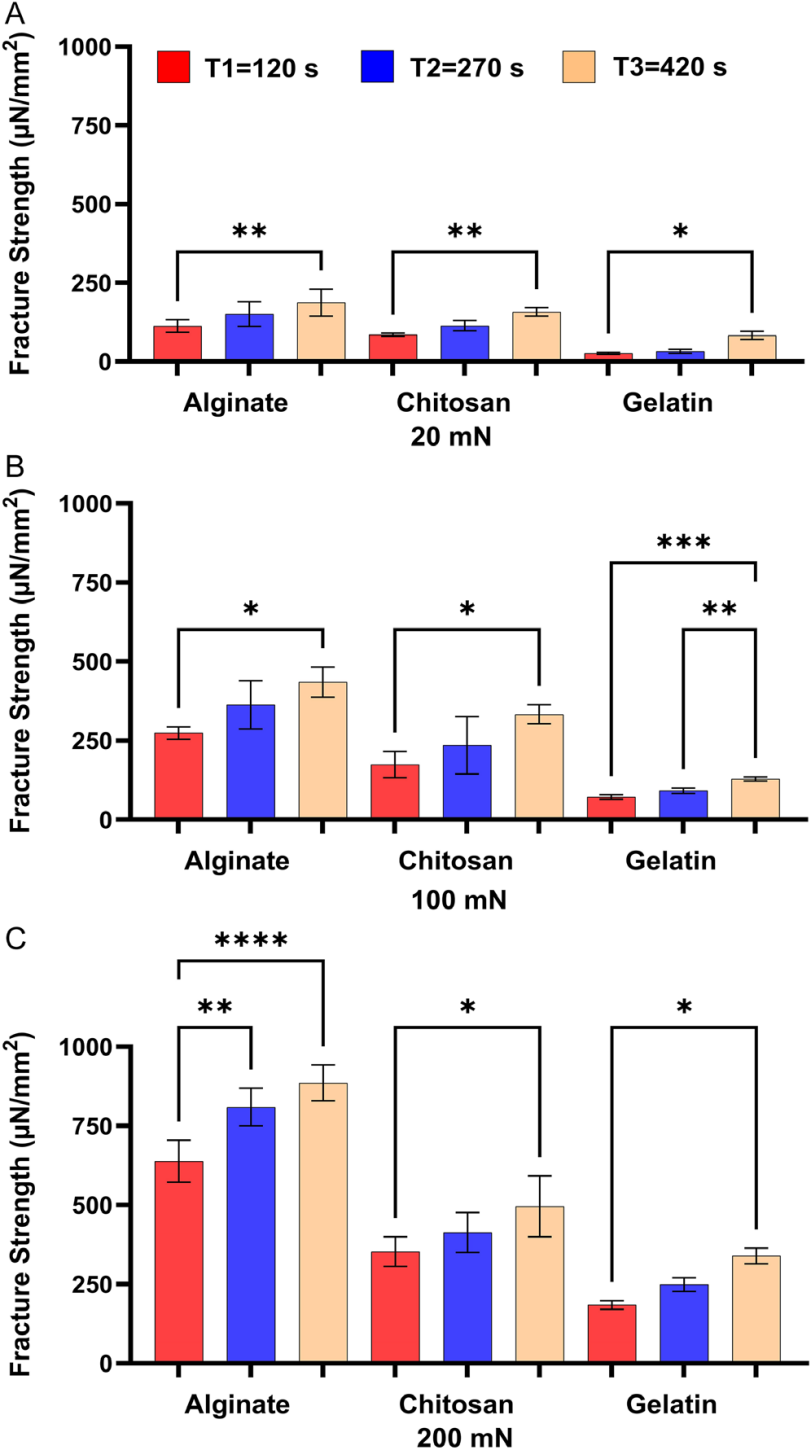
The effect of contact time on the bioadhesion of Alg, Chit, and Gel to the small intestines of sheep at the applied forces of A) 20 mN, B) 100 mN, and C) 200 mN. One‐way ANOVA was applied to determine statistical significance. Values represent the mean ± SD of *n* = 4; *p* values denoted as *p < 0.05, **p < 0.01, and ***p < 0.001.

**Figure 4:**
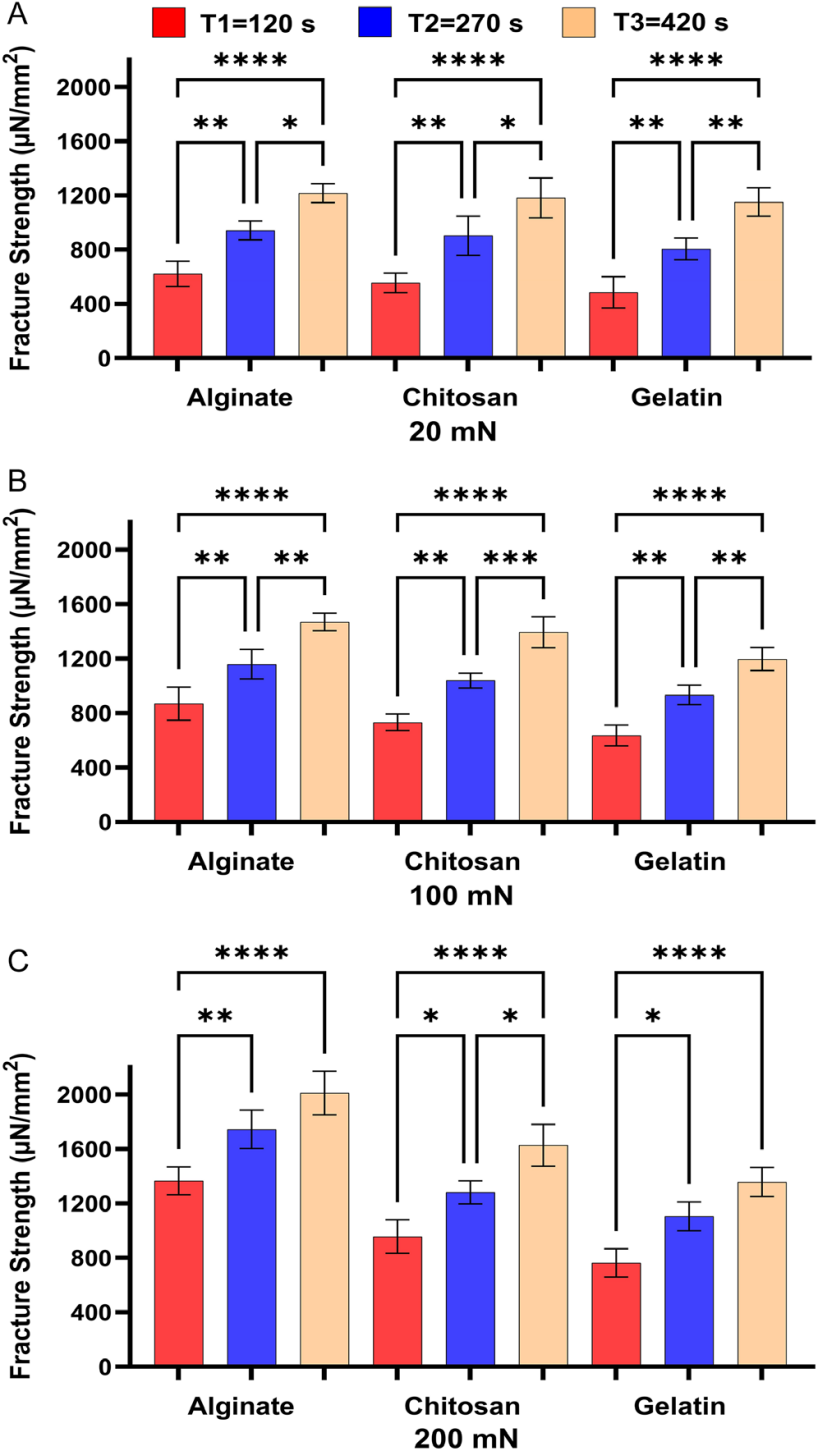
The effect of contact time on the bioadhesion of Alg, Chit, and Gel to the in vitro model at the applied forces of A) 20 mN, B) 100 mN, and C) 200 mN. One‐way ANOVA was applied to determine statistical significance. Values represent the mean ± SD of *n* = 4; *p* values denoted as *p < 0.05, **p < 0.01, and ***p < 0.001.

First, we assessed the effect of contact time on the varying degrees of bioadhesion bioadhesion properties of Alg, Gel, and Chit against the ex vivo small intestinal tissues of mice (**Figure** **1**), pigs (**Figure** **2**) (pigs), sheep (**Figure** **3**), and our cellular‐based in vitro model (**Figure** **4**).

The results shown in Figure 1, 2, 3, 4 show that under the tested applied forces (20–200 mN), there was a statistically significance increase in bioadhesion force for Alg, Chit, and Gel. This increase in bioadhesion with increased contact time was observed in all ex vivo and our in vitro models. More specifically, the most significant increase in bioadhesion was observed when the contact time was increased from 120 to 270 s. This trend was expected as it was shown before that prolonged contact time enables more complex bonding via mechanisms such as hydrogen bonding and electrostatic interactions.^[^ ^10^, ^29^ ^]^ In conclusion, our in vitro model (Figure 4) shows the same trend observed for the ex vivo models (Figure 1, 2, 3), affirming the model's ability to simulate natural biological responses over time. In the in vitro model, the cells were imaged before and after the test to check that Caco‐2 cells remained attached to Sylgard during the test (Figure 3S, Supporting Information).

### 2.2 Comparative Analysis of Applied Force Effect on Bioadhesion

**Figure 5:**
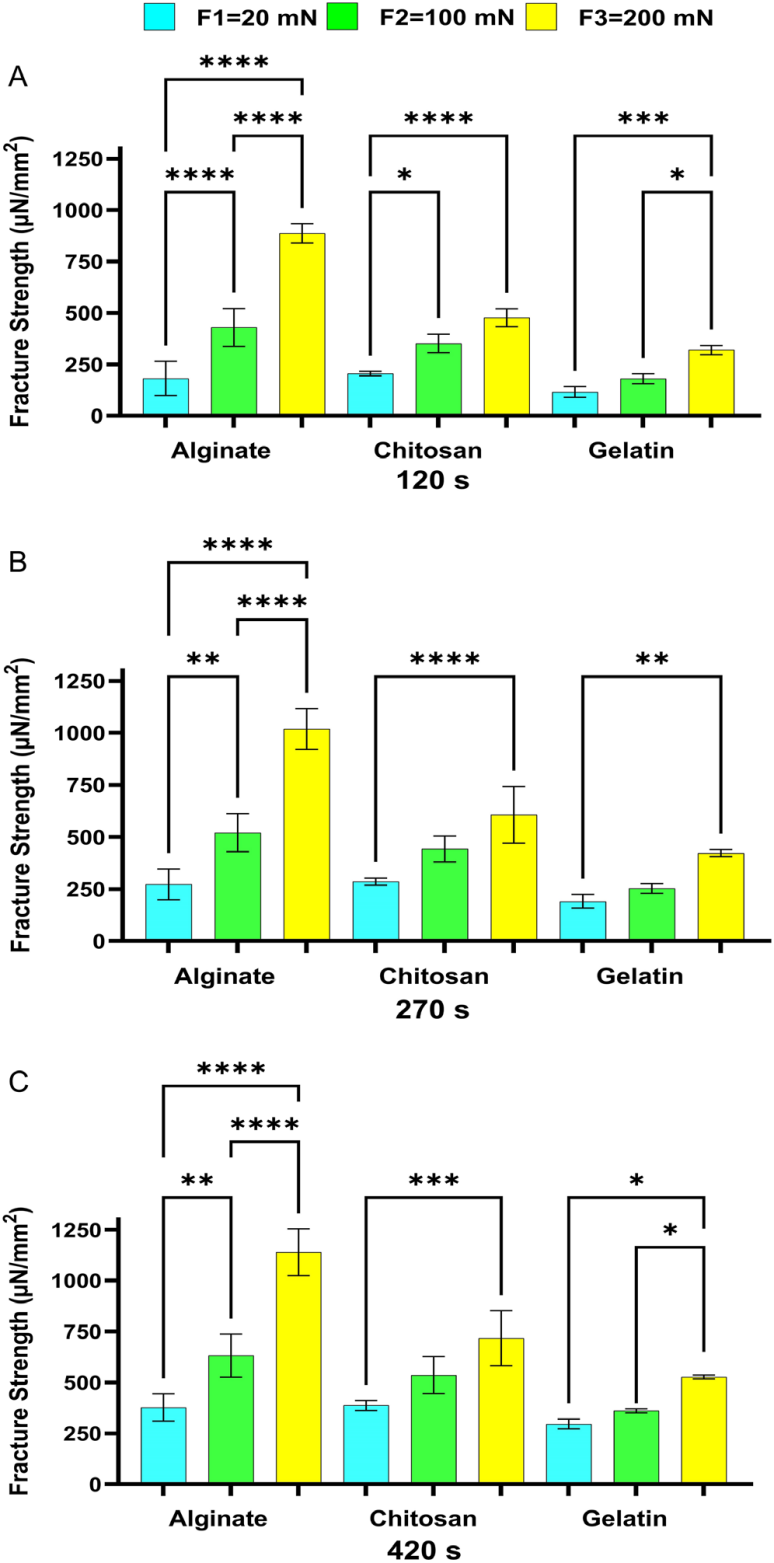
The effect of applied forces on the bioadhesion of Alg, Chit, and Gel to the small intestines of mice at the contact time of A) 120 s, B) 270 s, and C) 420 s. One‐way ANOVA was applied to determine statistical significance. Values represent the mean ± SD of *n* = 4; *p* values denoted as *p < 0.05, **p < 0.01, and ***p < 0.001.

**Figure 6:**
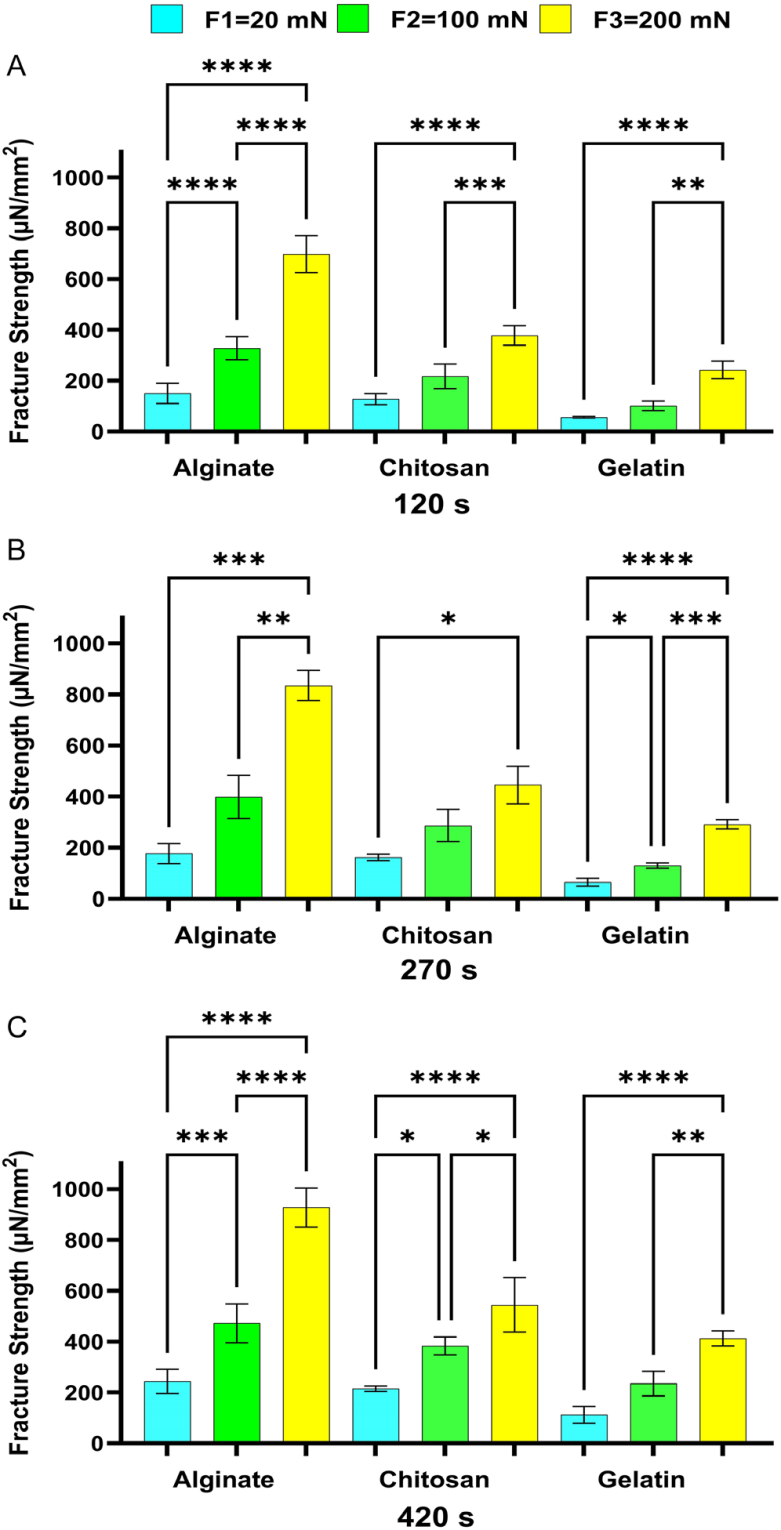
The effect of applied forces on the bioadhesion of Alg, Chit, and Gel to the small intestines of pigs at the contact time of A) 120 s, B) 270 s, and C) 420 s. One‐way ANOVA was applied to determine statistical significance. Values represent the mean ± SD of *n* = 4; *p* values denoted as *p < 0.05, **p < 0.01, and ***p < 0.001.

**Figure 7:**
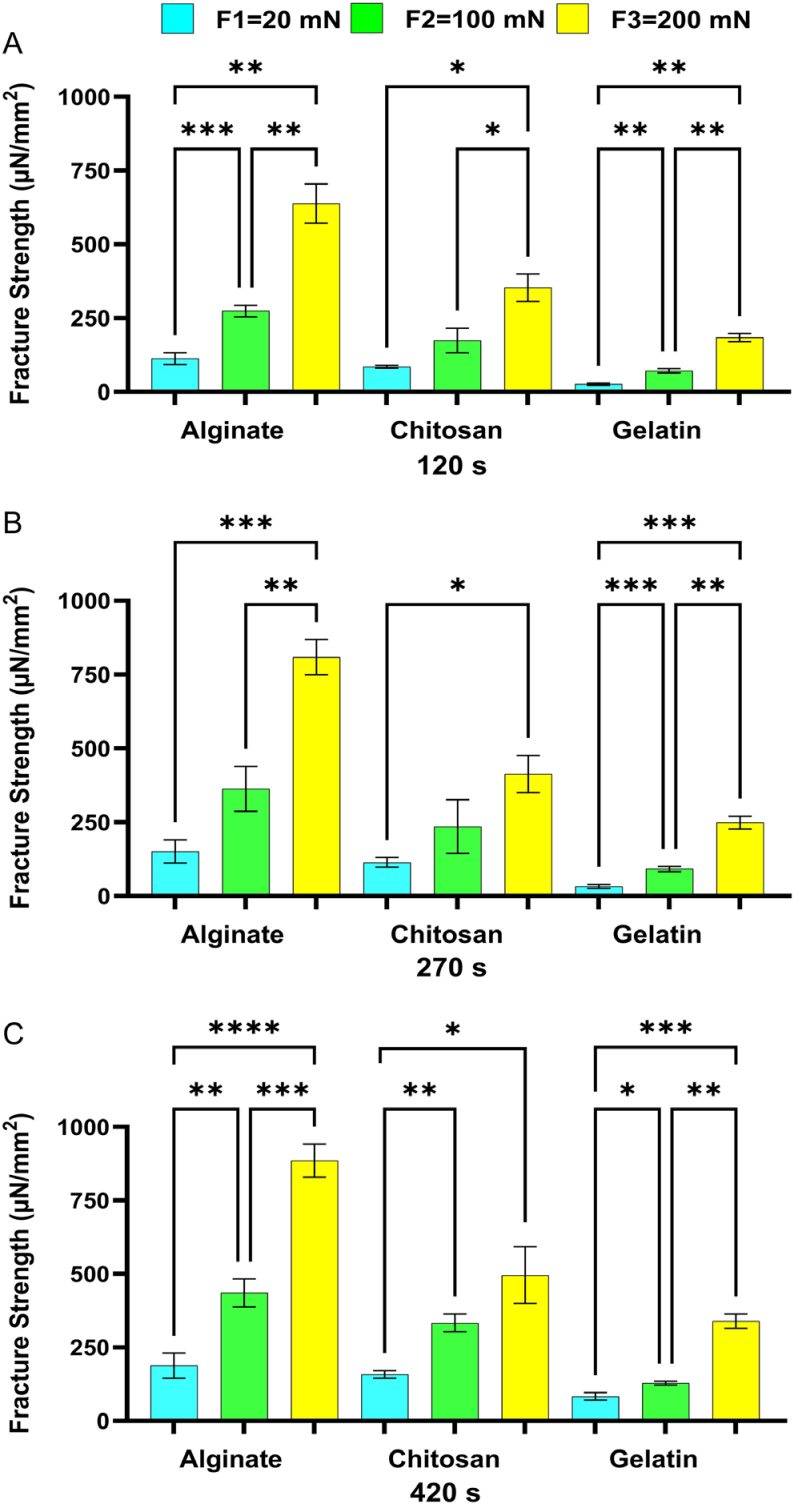
The effect of applied forces on the bioadhesion of Alg, Chit, and Gel to the small intestines of sheep at the contact time of A) 120 s, B) 270 s, and C) 420 s. One‐way ANOVA was applied to determine statistical significance. Values represent the mean ± SD of *n* = 4; *p* values denoted as *p < 0.05, **p < 0.01, and ***p < 0.001.

Continuing our examination of bioadhesion properties, we assess how the applied force attaching the two surfaces/materials affects the bioadhesion fracture strengths of Alg, Chit, and Gel. **Figure** **5**, **6**, **7**, **8** present the statistical analysis performed to analyze this effect.

**Figure 8:**
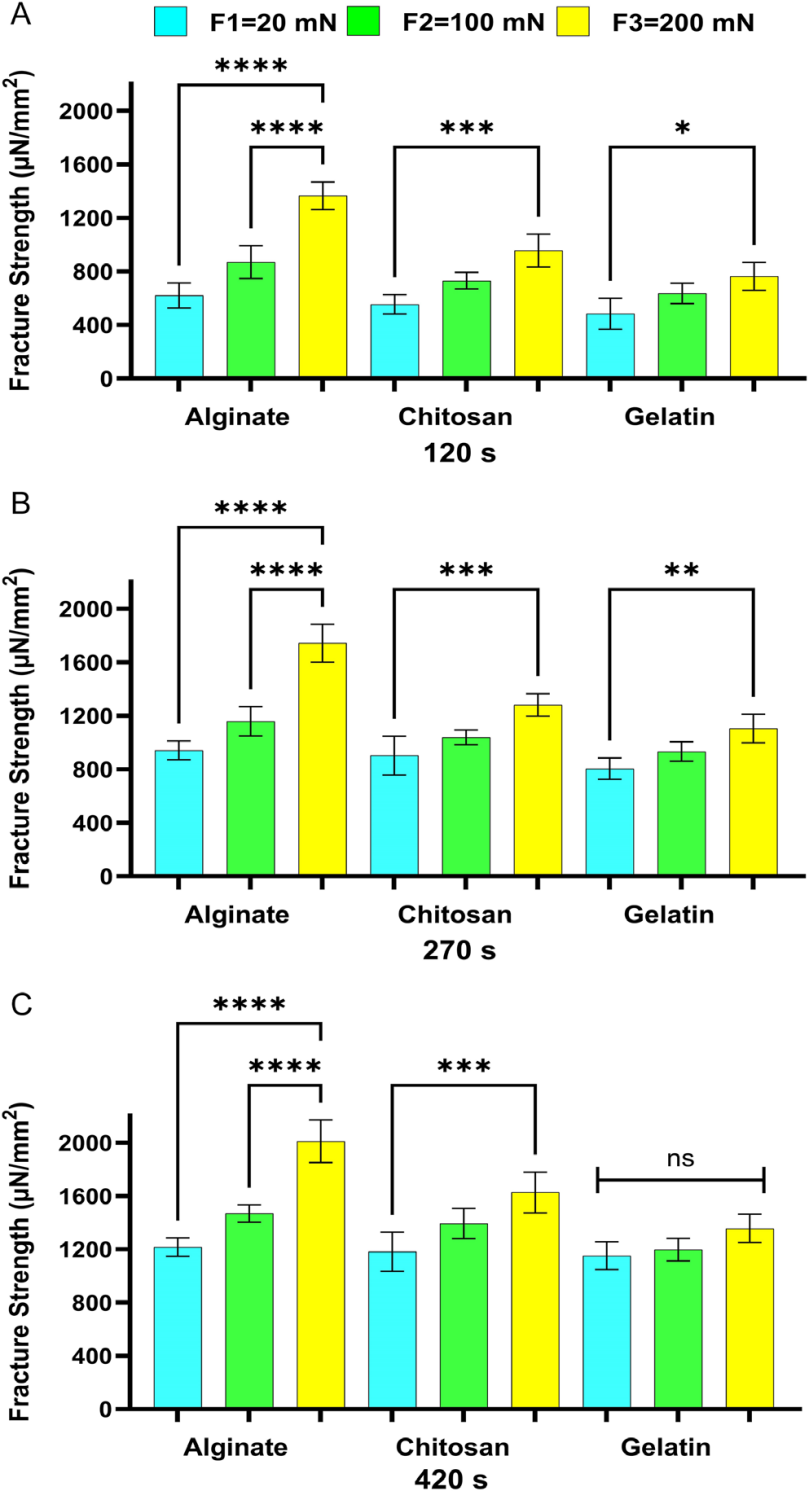
The effect of applied forces on the bioadhesion of Alg, Chit, and Gel to the in vitro model at the contact time of A) 120 s, B) 270 s, and C) 420 s. One‐way ANOVA was applied to determine statistical significance. Values represent the mean ± SD of *n* = 4; *p* values denoted as *p < 0.05, **p < 0.01, ***p < 0.001, and ns—nonsignificant.

The comparisons in Figure 5 through 8 exhibit a similar influence as contact time on bioadhesion fracture strengths. Generally, the bioadhesion increased as the applied force increased the bioadhesion in the ex vivo models—mice, pigs, and sheep (Figure 5, 6, 7)—and in the in vitro model (Figure 8). This trend was expected as it was shown before that prolonged contact time enables more complex bonding via hydrogen bonding and electrostatic interactions.^[^ ^10^, ^30^ ^]^ This trend was observed for tested bioadhesives—Alg, Chit, and Gel. In conclusion, our in vitro model exhibits the same effect of increased applied force on bioadhesion as observed for the ex vivo models, further affirming the model's ability to simulate ex vivo tissue responses.

### 2.3 Comparative Evaluation of Materials Effect on Bioadhesion

Continuing our evaluation of our in vitro model's ability to predict and measure intestinal bioadhesion fracture strength, we next analyzed the ability of our in vitro model to predict which material (i.e., Alg, Chit, and Gel) is more bioadhesive under different contact times and applied forces. To this end, we summarized the bioadhesive fracture strength order of the materials per model and specific contact time and applied force (**Table** **1**).

**Table 1:** Comparative analysis of Alg, Chit, and Gel's bioadhesive fracture strengths in the ex vivo vs. the in vitro models. ‘F’ – applied forces of F1 = 20 mN, F2 = 100 mN, and F3 = 200 mN. ‘T’ – contact times of T1 = 120 s, T2 = 270 s, and T3 = 420 s). Statistical difference was deemed at *p < 0.05 and ns—nonsignificant.

Test parameters	In vitro model	Ex vivo mouse	Ex vivo pig	Ex vivo sheep	Match to in vitro model
F1, T1	ns	ns	ns	Alg = Chit > Gel	Mouse/Pig
F1, T2	ns	ns	Chit > Gel	Chit > Gel	Mouse
F1, T3	ns	ns	Chit > Gel	Chit > Gel	Mouse
F2, T1	Alg > Gel	Alg > Gel	ns	Alg > Gel	Mouse/Sheep
F2, T2	Alg > Gel	Alg = Chit > Gel	ns	ns	Mouse
F2, T3	Alg > Gel	Alg > Gel	ns	Alg = Chit > Gel	Mouse/Sheep
F3, T1	Alg > Chit = Gel	Alg > Chit = Gel	Alg > Chit = Gel	Alg > Chit > Gel	Mouse/Pig/Sheep
F3, T2	Alg > Chit = Gel	Alg > Chit = Gel	Alg > Chit = Gel	Alg > Chit > Gel	Mouse/Pig/Sheep
F3, T3	Alg > Chit = Gel	Alg > Chit = Gel	Alg > Chit = Gel	Alg > Chit > Gel	Mouse/Pig/Sheep

Table 1 outlines the bioadhesive strengths of each material under varying conditions, providing a direct analysis of their comparative effectiveness in both ex vivo and in vitro models. In this table, a material is defined as having higher/lower bioadhesion than another material when *p* < 0.05. Materials that did not show any statistically significant difference are not displayed. The last column (Match to in vitro model) examined the trends between the substances obtained in the ex vivo models and those obtained in the in vitro model.

As shown in Table 1, in most cases, the order of bioadhesion strength across the ex vivo models was Alg > Chit > Gel. This hierarchy was especially evident when the applied force was 200 mN and, to a lesser extent, at 100 mN. Notably, at higher applied forces (100 and 200 mN), the in vitro model exhibited the same order and provided a more apparent distinction between these materials. Thus, we postulate that at 20 mN, there is an increased variability in the results since the applied contact force is too weak to ensure complete contact between the two surfaces. Furthermore, the results in Table 1 strongly indicate that our in vitro model is most comparable to the ex vivo mouse model, showing a 100% match. Mice are generally recognized as the most suitable animal model for oral drug delivery research due to their similarity to humans’ physiological and genetic characteristics,^[^ ^31^ ^]^ mass transport properties,^[^ ^32^ ^]^ including similarity in the mucin sequence.^[^ ^24^ ^]^ On the contrary, the pig and sheep animal models were ≈50% similar to the in vitro model. However, it should be noted that at an applied force of 200 mN, all models demonstrated a 100% match. The observed differences in bioadhesion fracture strength in the three ex vivo animal tissue models are discussed separately in Section 3.5.

Alginate was simply one of the biomaterials we used to test our model for its bioadhesive properties. Its inclusion in this study was intended to enable a comparison of the bioadhesion fracture strength among different well‐known bioadhesive biomaterials, specifically chitosan and gelatin. This comparison allowed us to evaluate the effectiveness of each biomaterial in promoting bioadhesion within our model, providing insights into the relative bioadhesive strengths across materials commonly used in drug delivery applications.

### 2.4 Predicting Absolute Bioadhesion Values

Once we have established the ability of our model to predict the effects of contact time, applied force, and materials on intestinal bioadhesion, we compared the absolute values obtained by our in vitro model to those obtained by the ex vivo models. To this end, we divided the values obtained from the ex vivo model by the respective values obtained from the ex vivo models. The averaged values per material and model are the bioadhesion fracture strength ratios presented in **Table** **2**.

**Table 2:** In vitro versus ex vivo models (i.e., mouse, pig, and sheep) bioadhesion fracture strength ratios (vs the in vitro model) for Alg, Chit, and Gel.

Ex vivo model	Chit ratio	Alg ratio	Gel ratio
Mouse	2.5 ± 0.4	2.4 ± 0.8	3.4 ± 0.7
Pig	3.8 ± 1.1	3.3 ± 1.3	6.7 ± 3.2
Sheep	4.9 ± 1.9	3.8 ± 1.8	10.9 ± 7

The results shown in Table 2 reveal that the bioadhesion fracture strength measured in our in vitro model was consistently higher compared to the corresponding measurements in ex vivo models. Specifically, our in vitro model showed the closest alignment with mice ex vivo intestinal tissues (followed by porcine and sheep models) with bioadhesion ratios of 2.4 to 3.4‐fold compared to the in vitro model. Among the materials tested, Gel exhibited the highest bioadhesion ratios of 3.4 to 10.9‐fold, while Alg exhibited the smallest ratios, ranging from 2.4 to 3.8‐fold. Notably, under a force of 200 mN, Alg ratios remained relatively stable, with 1.5 to 1.8‐fold for the mouse model, 2 to 2.2‐fold for the pig model, and 2.1 to 2.3‐fold for the sheep model (Table 1S, Supporting Information). In contrast, Gel bioadhesion ratios were 3.4‐fold for mice, 6.7‐fold for pigs, and 10.9‐fold for sheep. Generally, the bioadhesion ratios of the ex vivo tissues compared to the in vitro model were smaller at higher applied forces, further supporting our hypothesis that higher applied forces ensure better contact between the two surfaces.

### 2.5 The Effect of the Morphology of Intestinal Microvilli on Bioadhesive

One surprising insight from the comprehensive analysis was that bioadhesion fracture strength values varied significantly between animal models. As mentioned, these models are used routinely to measure bioadhesion (especially porcine and mice^[^ ^24^ ^]^). Therefore, it is imperative to understand why there are such differences between animal models to improve in vitro/ex vivo/in vivo correlation. Thus, herein, we attempt to explain this observation. We hypothesize that since bioadhesion is a surface property, the models’ differences relate to the intestines’ microvilli morphology.

To understand the influence of intestinal morphology on bioadhesive fracture strength, we conducted a detailed image analysis of the intestinal microvilli characteristics of the tested animal models. To this end, we have defined two critical parameters: microvilli linear density and microvilli width. These parameters were measured and calculated using ImageJ's scale bar measurement tools. The results of the microvilli morphology analysis are summarized in **Table** **3**.

**Table 3:** Intestinal microvilli morphology of humans, Caco‐2 cells, mice, pigs, and sheep, measured from histological images using ImageJ (Figure 5S, Supporting Information).

	Microvilli linear density [microvilli mm^−1^]	Width of microvilli [mm]
Human	5.1	0.16
Caco‐2 cells	6.2	0.14
Mouse	10.3	0.05
Pig	12.8	0.07
Sheep	14.5	0.06

As can be seen in Table 3, Caco‐2 cells have a lower microvilli density (6.2 microvilli mm^−1^) compared to mice (10.3 microvilli mm^−1^), pigs (12.8 microvilli mm^−1^), and sheep (14.5 microvilli mm^−1^). The reduction in microvilli density also correlates with reduced bioadhesion fracture strength. Hence, there is an inverse relationship between microvilli density and bioadhesion; the higher the microvilli density is, the lower the measured bioadhesion. The inverse relationship between microvilli density and bioadhesion fracture strength could be attributed to increased mucin interaction stemming from the lower microvilli density. As for the width of the microvilli, there was no correlation to bioadhesion fracture strength.

This observation can explain why higher bioadhesion values were observed in our Caco‐2‐based in vitro model compared to the animal ex vivo models and why mice tissues were closer in their bioadhesion values compared to pigs and sheep tissues. Moreover, the similarity of Caco‐2 cells to human intestinal tissue in microvilli density (6.2 vs 5.1 microvilli mm^−1^, respectively) reinforces their relevance in oral drug delivery research. In conclusion, the correlation between microvilli density and bioadhesion further solidifies the ability of our in vitro model to serve as a reliable (and potentially more accurate) alternative to ex vivo animal tissues for the measurement of bioadhesion fracture strength.

## 3 Conclusions

The in vitro Caco‐2‐based bioadhesion model was developed to simulate the human intestinal epithelium, offering significant ethical and scientific advantages by reducing reliance on animal testing and potentially increasing similarity to humans. Our in vitro model predicted the observed effects in the ex vivo models of increased contact time and applied force on the bioadhesion property of common bioadhesives (i.e., Chit, Alg, and Gel). Both the in vitro and ex vivo models showed that when contact time and applied forces are increased, so is the measurement of bioadhesion fracture strength. Moreover, our in vitro model could predict which material was more bioadhesive (as demonstrated in the ex vivo models), further supporting its applicability for testing and comparing bioadhesives used in oral drug delivery.

These findings will provide researchers in the field of bioadhesive DDS with a practical tool to better assess and compare bioadhesive materials, ultimately aiding in the development of more effective DDS. Our in vitro model provides a practical, widely accessible, and cost‐effective tool for researchers in the field of bioadhesive DDS. It enables precise assessment of bioadhesion properties essential for designing effective delivery systems. By offering a reproducible alternative to ex vivo models, this approach supports the replacement, reduction, and refinement of animal tissue usage in bioadhesive research and has the potential to be integrated into standardized testing protocols. Consequently, our model aids in advancing bioadhesive DDS development by offering enhanced applicability and ethical benefits.

Furthermore, the study revealed a significant difference in the measured bioadhesion values between the three animal ex vivo tissue models of sheep, mice, and pigs. Generally, it was observed that mice tissues exhibited the highest bioadhesion properties, followed by porcine and sheep tissues. We found that this phenomenon could be explained by the inverse relationship between microvilli linear density and bioadhesive strength, emphasizing the importance of considering intestinal morphology in developing effective bioadhesive DDS. Moreover, our in vitro model is the closest in microvilli linear density to human intestines, suggesting it could be even more accurate in predicting human bioadhesion than the ex vivo animal tissues. This is particularly important as human tissues are rarely available. Furthermore, the study established that bioadhesion could be reliably measured up to 7 min, beyond which drying of the biopolymers became apparent. Although we tested contact durations up to 10 min, the observed drying effect limited further bioadhesive assessment, underscoring a practical constraint of our method. In conclusion, our model and findings could be beneficial for researchers in the field of bioadhesive and general oral DDS as it provides an in vitro alternative to measuring bioadhesion, improves in vitro‐in vivo correlation, and provides essential insights about the effect of intestinal morphology (i.e., microvilli linear density) on bioadhesion. While this study focuses on measuring bioadhesion fracture strength, the insights gained provided a foundation for future developments of more complex in vitro models, such as 3D bionic intestinal mucosa systems. These advanced models, which could replicate additional aspects of intestinal physiology, would enable a more comprehensive study of DDS and mucosal interactions in a controlled laboratory setting. Our Caco‐2‐based model has the potential to contribute to this goal by mimicking the surface properties of the GI tract, possibly through shaping the SYLGARD layer to reflect a microvilli‐specific structure.

## 4 Experimental Section

4.1

4.1.1

##### Materials

Caco‐2 cells were obtained from the American Type Culture Collection (ATCC HTB‐37). Culture media components, including Dulbecco's Modified Eagle Medium (DMEM), L‐Glutamine (L‐Glu), Penicillin–Streptomycin (P/S), phosphate‐buffered saline (PBS), and fetal bovine serum (FBS), were procured from Sartorius, Israel. Sodium alg, gel, and chit were purchased from Sigma‐Aldrich, Israel. Sylgard 184 silicone elastomer was obtained from Dow Corning, USA (supplied by Polymer G, Israel).

##### Hydrogels Preparation

Hydrogels of sodium alg, gel, and chit were prepared daily before experiments. Alg and gel were prepared at 5% w/v in Milli‐Q water, while chit was prepared at 2% w/v chit in a 1% v/v acetic acid aqueous (Milli‐Q water) solution.

##### In vitro Model Preparation

SYLGARD 186 silicone elastomer was prepared in a 10:1 ratio (base:curing agent, respectively) and applied to coat Petri dishes (9 cm Romical, Israel). The elastomer was spread to create a uniform layer with a height of 3 mm and was allowed to solidify at room temperature for three days. Petri dishes with sylgard were exposed to UV for 1 h for sterilization. Growing cells on PDMS surfaces is challenging due to its intrinsic hydrophobicity. Hydrophobicity might lead to limited wettability and reduced cell adhesion on PDMS surfaces, as these surfaces do not inherently support strong cell attachment.^[^ ^33^, ^34^ ^]^ In our model, we addressed this challenge by extending the culture period to achieve stable adherence. After the solidification of the sylgard, Caco‐2 cells were seeded at a density of 5 × 10^5^ cells mL^−1^ (7900 cells cm^−2^) and incubated at 37 °C with 5% CO_2_. Achieving full confluency on the PDMS surface required 5–8 days, with regular medium replacements to optimize cell growth. Once confluency was attained, the cell layer was cut into squares with a surface area of 25 mm^2^ for subsequent bioadhesion tests.

SYLGARD 186 was selected as the substrate material in our bioadhesion model due to its ability to securely attach to the mounting pin, a crucial feature for ensuring consistency in bioadhesion measurements, which hydrogel‐based substrates do not provide. UV light exposure can induce the formation of a SiO_2_‐like layer on the PDMS surface, known for its enhanced hydrophilicity, which may facilitate cell adhesion.^[^ ^35^, ^36^ ^]^ During the UV sterilization of our samples, this modification likely improved the PDMS surface properties, enabling more effective cell adherence. This insight suggests that UV‐treated PDMS may serve as a viable substrate for bioadhesion studies involving cell cultures.

##### Ex vivo Tissues Preparation

As mentioned, tissues from the small intestines of mice, pigs, or sheep were used to validate our in vitro model. Ex vivo intestinal tissues were prepared by first washing them with PBS. These tissues are used either immediately or after freezing at −20 °C for a maximum of three days (a test was made in the model system of fresh tissue versus tissue frozen for three days, and it was found that there is no difference in the values obtained). The tissues were cut to an approximate square area of 40 mm^2^ to ensure consistent sample size across experiments.

##### Bioadhesion Testing

Bioadhesion testing was conducted using a Shimadzu EZ‐SX texture analyzer. Our customized method was customized for soft matters, as shown before.^[^ ^37^ ^]^ First, we used a flat pin on the analyzer's probe to support the tissue sample or the in vitro model. Each hydrogel formulation, 50 μL in volume, was applied to a glass slide, which served as the carrier. The glass slide was positioned directly under the mounted pin, with the testing areas set to 25 mm^2^ for the in vitro model and 40 mm^2^ for the ex vivo tissues. The texture analyzer's probe descended at a speed of 1.0 mm s^−1^, applying forces of F1 = 20 mN, F2 = 100 mN, and F3 = 200 mN for durations of T1 = 120 s, T2 = 270 s, and T3 = 420 s. We measured the peak force needed to detach the hydrogel from the mucosal surface (Figure 1S, Supporting Information). This force was normalized by the contact area to determine the bioadhesion strength per unit area, providing a quantitative measure of the hydrogel's adhesion capabilities (Figure 2S, Supporting Information). A more detailed protocol can be found in the Supporting Information file.

##### Microvilli Morphology Analysis

Histological images of intestinal tissues from humans,^[^ ^38^ ^]^ mice,^[^ ^39^ ^]^ pigs,^[^ ^40^ ^]^ sheep,^[^ ^25^ ^]^ and Caco‐2 cells^[^ ^41^ ^]^ were analyzed to determine microvilli morphology parameters, density (microvilli mm^−1^), and width (mm) to evaluate their effect on bioadhesion. The images were analyzed using ImageJ software (version ij154). The analysis was done using the scale bar measurement tools, and according to the scale bar, all the microvilli linear density and width were measured and calculated.

##### Statistical Analysis

GraphPad Prism version 10 was used to analyze the data. The Shapiro–Wilk test was applied to check data normality, while the Brown–Forsythe test assessed the homogeneity of variances. Group differences were evaluated using a one‐way ANOVA analysis with Fisher's Least Significant Difference (LSD) post hoc test at *α* = 0.05. For data that exhibited a difference in variances, the BrownForsythe one‐way ANOVA test was used. Presented values are the mean ± standard deviation (SD) of a minimum of *n* = 4 per condition.

## Conflict of Interest

The authors declare no conflict of interest.

## Author Contributions

**Eliyahu Drori**: methodology, data curation, validation, formal analysis, writing—original draft, preparation. **Valeria Rahamim**: review, data curation, and editing. **Dhaval Patel**: review and validation. **Yamm Anker**: review and validation. **Sivan Meir**: review and validation. **Gal Uzan**: data curation. **Shira Somech**: review and validation. **Chen Drori**: statistical analysis. **Tal Tzadok**: statistical analysis. **Aharon Azagury**: scientific integrity, conceptualization, writing, editing, and project supervisor. All authors have read and agreed to the published version of the manuscript.

## Supporting information

Supplementary Material

## Data Availability

The data that support the findings of this study are available from the corresponding author upon reasonable request.
