# Supplementary material for: An In vitro Caco2‐Based Model for Measuring Intestinal Bioadhesion Comparable to Ex vivo Models
Source: Small Sci. 2024 Dec 3;5(2):2400461. doi: 10.1002/smsc.202400461 (PMC11934890; doi:10.1002/smsc.202400461)
Supplement: Supplementary file 1 — Supplementary Material [file SMSC-5-2400461-s001.pdf]

Supporting Information

An *In Vitro*, Caco2-Based Model for Measuring Intestinal Bioadhesion Comparable to *Ex Vivo* Models

By: Eliyahu Drori[1], Valeria Rahamim[1], Dhaval Patel[1], Yamm Anker[1], Sivan Meir[1], Gal Uzan[1], Shira Somech[2], Chen Drori[1], Tal Tzadok[3], and Aharon Azagury[1]*

[1]Department of Chemical Engineering and Biotechnology, Ariel University, Kiryat Hamada 3, Ariel, Israel

[2]Department of Medical and Health Sciences - Tel Aviv University, Klachkin 35, Tel Aviv-Yafo, Israel

[3]Department of Maurice and Gabriela Goldschleger School of Dental Medicine - Tel Aviv University, Klausner 6, Tel Aviv-Yafo, Israel

*Corresponding author: aharona@ariel.ac.il

Detailed Protocol for Preparing Bioadhesion Fracture Strength *In Vitro* Model

1. PDMS Preparation

1. Prepare the PDMS mixture by combining Sylgard™ 186 base and curing agent in a 10:1 mass ratio, following the manufacturer's instructions to ensure the desired elasticity and mechanical stability.

2. Pour the PDMS mixture into 9 cm diameter Petri dishes, creating a uniform layer of approximately 3 mm thickness.

3. Tap the Petri dishes gently to release any air bubbles trapped in the PDMS mixture, as bubbles could interfere with cell attachment or surface smoothness.

4. Allow the PDMS to solidify at room temperature for 72 h, covering the dishes to protect them from dust and contaminants.
5. Sterilize the PDMS-coated dishes by exposing them to UV light for 1 h before proceeding with cell seeding.

2. Cell Culture Work

1. Prepare Caco-2 cells by culturing them under standard conditions (37°C, 5% CO_2) in Dulbecco's Modified Eagle Medium (DMEM) supplemented with L-Glutamine, Penicillin-Streptomycin, and fetal bovine serum (FBS).
2. Seed Caco-2 cells on the sterilized PDMS-coated Petri dishes at a density of $5x10^5$ cells/mL (7900 cells/cm^2).
3. Incubate the cells at 37°C with 5% CO_2, replacing the culture medium every two days to remove metabolic waste products and provide fresh nutrients essential for promoting healthy cell proliferation.
4. Monitor cell confluency visually using an inverted microscope until a continuous monolayer is achieved across the PDMS surface, approximately within 5 to 8 days.
5. Cut the monolayer into squares (0.5 cm wide) with a surface area of 25 mm^2 once full confluency is reached. These squares will be used in subsequent bioadhesion tests, ensuring consistency in size and thickness.

3. Sample Preparation

1. Prepare alginate (Alg)/gelatin (Gel) hydrogels by weighing 2.5 g of the sample and dissolving it in 50 mL of Milli-Q water, creating a 5% (w/v) hydrogel solution.

2. Mix the solution at room temperature and stir continuously until complete dissolution.
3. Remove any bubbles from the hydrogel solution to ensure sample uniformity.
4. Store the hydrogel solution at room temperature in a scintillation vial until use.
5. Prepare chitosan hydrogel by dissolving 1.0 g of chitosan in 50 mL of a 1% (v/v) acetic acid aqueous solution, creating a 2% (w/v) hydrogel solution. Stir and heat gently, if necessary, to fully dissolve the chitosan, as it tends to aggregate in solution.
6. Prepare *ex vivo* tissue samples by collecting small intestine tissues from mice, pigs, or sheep under sterile conditions.
7. Place the collected tissues on ice immediately after collection and transport them to the laboratory.
8. Thaw and rinse the tissues with phosphate-buffered saline (PBS) to remove blood and other debris before use.
9. Cut the tissues into squares of approximately 40 mm^2 using sterile surgical scissors, ensuring consistency across different animal species.
10. Store tissues at -20°C for up to three days if not used immediately, following standard procedures for preserving tissue integrity. Allow tissues to equilibrate to room temperature before testing.

4. Texture Analyzer Protocol

1. Mount the tissue/PDMS squares onto the flat pin probe of the Shimadzu EZ-SX texture analyzer using super glue.

2. Pipette 50 µL of hydrogel formulation (Alg, Gel, or Chit) onto a glass slide, serving as the hydrogel carrier.
3. Position the glass slide on the bottom platform of the texture analyzer.
4. Set the texture analyzer to lower the probe at a controlled speed of 1.0 mm/s until it contacts the hydrogel sample.
5. Upon contact, apply the desired force (e.g., 20 mN, 100 mN, and 200 mN).
6. Allow the surfaces to interact for the desired contact durations (e.g., 120 s, 270 s, and 420 s).
7. Record the peak detachment force required to separate the hydrogel from the mucosal surface (either *in vitro* or *ex vivo*). This force, known as the peak detachment force, is normalized by the contact area to calculate bioadhesion strength per unit area.

Following the above protocol, a sample of the texture analyzer output is presented in **Figure 1S**.

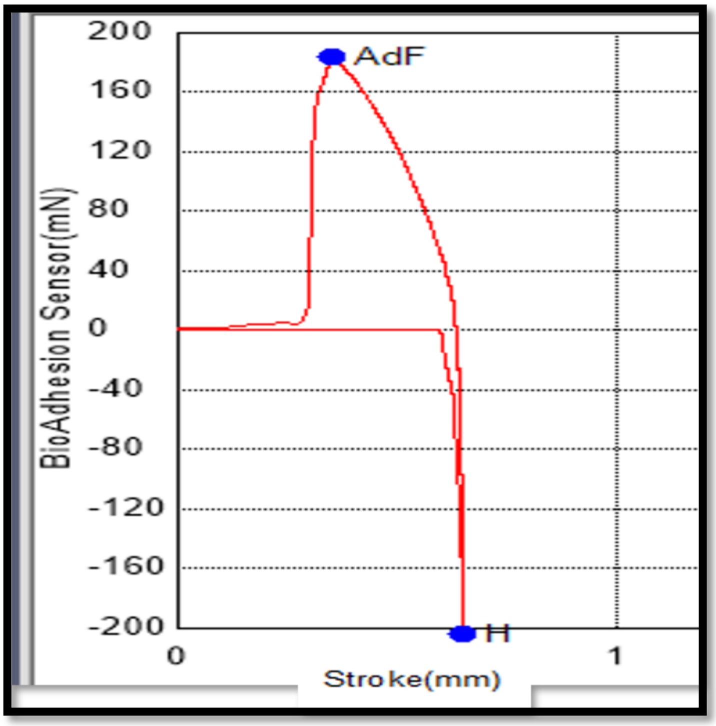

Figure 1S. Force-distance profile during bioadhesion testing

Figure 1S represents the recorded data from the bioadhesion sensor force in a graphical format of the bioadhesion force versus the vertical movement of the probe during bioadhesion testing. The curve begins at the baseline, with the initial contact between the probe-mounted tissue or *in vitro* model and the partially dried hydrogel on the carrier glass slide. The blue point H indicates the applied force (e.g., herein, a force of 200 mN was applied for 420 s). Then, the pin is pulled until the two surfaces detach (indicated by the blue point labeled as AdF. This peak force illustrates the maximum detachment force necessary to separate the tissue/sylgard from the tested material. Then, this peak force is divided by the specific surface of the tissue/sylgard sample.

The application of the method using the texture analyzer is shown in **Figure 2S** below.

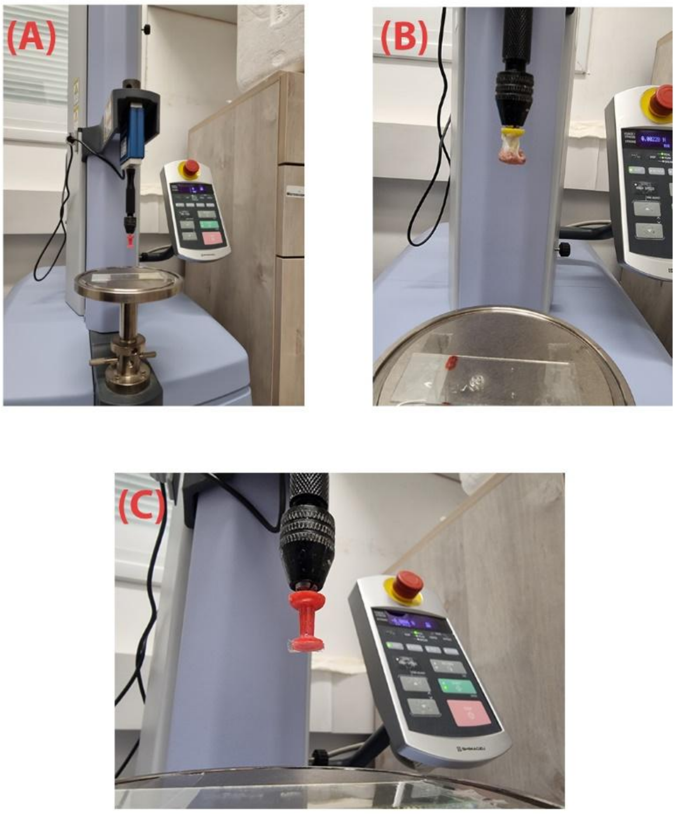

Figure 2S. Setup of the texture analyzer for bioadhesion fracture strength testing.

Figure 2S illustrates the texture analyzer utilized to evaluate the bioadhesion properties of hydrogels. **Figure 2**SA shows the apparatus with the probe extending downwards, where a red pin is affixed. The red pin serves as the mounting point for the tissue or sylgard sample. Positioned on the base is a carrier glass holding the tested hydrogel sample. **Figure 2S B** captures the setup with pig intestinal *ex vivo* tissue secured to the end of the probe pin, ready for the bioadhesion test. **Figure 2S C** displays a section of Sylgard affixed to the probe pin, with a confluent layer of Caco-2 cells oriented downwards toward the hydrogel on the carrier glass before the (*in vitro*) bioadhesion evaluation.

An image was taken under a microscope to test the tissue capacity of caco-2 cells attached to the sylgard and to determine if they could withstand

the load applied by the tissue analyzer during the bioadhesion cycle, as shown in **Figure 3S**.

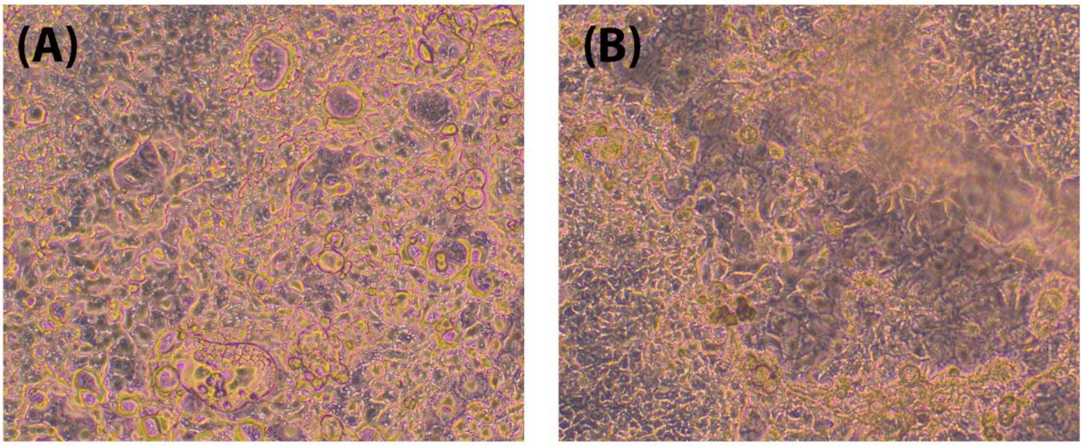

Figure 3S. Microscopic examination of caco-2 cells pre- and post-adhesion test.

Figure 3S presents microscopic images of caco-2 cells used in the bioadhesion test. **Figure 3S A** displays the cells on sylgard prior to the measurement by the texture analyzer, with cells appearing well-adhered and evenly distributed across the surface in full confluency. **Figure 3S B** shows the same cells on sylgard after the bioadhesion test, where a force of 200 mN was applied for 420 seconds. Despite the significant force exerted over an extended duration, the cells demonstrate no notable detachment, indicating the test measured the bioadhesion between the cells and the tested material.

Table 1S. *In vitro* and *ex vivo* bioadhesion strength ratios compare the bioadhesive bond strengths of alginate, chitosan, and gelatin. Here, 'F' designates the levels of force applied (F1 = 20 mN, F2 = 100 mN, F3 = 200 mN), and 'T' specifies the application durations (T1 = 120 s, T2 = 270 s, T3 = 420 s).

***Ex vivo* model**	**Factors**	**Chitosan Ratio**	**Alginate Ratio**	**Gelatin Ratio**
Mouse	F1,T1	2.7	3.4	4.2
	F1,T2	3.2	3.5	4.2
	F1,T3	3.1	3.2	3.9

	F2,T1	2.1	2.0	3.5
	F2,T2	2.3	2.2	3.7
	F2,T3	2.6	2.3	3.3
	F3,T1	2.0	1.5	2.4
	F3,T2	2.1	1.7	2.6
	F3,T3	2.3	1.8	2.6
Pig	F1,T1	4.3	4.1	8.6
	F1,T2	5.6	5.3	12.3
	F1,T3	5.5	5.0	10.3
	F2,T1	3.4	2.6	6.3
	F2,T2	3.6	2.9	7.2
	F2,T3	3.6	3.1	5.1
	F3,T1	2.5	2.0	3.1
	F3,T2	2.9	2.1	3.8
	F3,T3	3.0	2.2	3.3
Sheep	F1,T1	6.5	5.5	18.7
	F1,T2	7.9	6.2	24.4
	F1,T3	7.5	6.5	13.8
	F2,T1	4.2	3.2	8.9
	F2,T2	4.4	3.2	10.2
	F2,T3	4.2	3.4	9.3
	F3,T1	2.7	2.1	4.1
	F3,T2	3.1	2.2	4.4
	F3,T3	3.3	2.3	4.0

Table 1S illustrates the comparative strength ratios of bioadhesive bonds formed by alginate, chitosan, and gelatin in both *in vitro* and *ex vivo* models. These ratios help in understanding the relative performance and effectiveness of the biopolymers. The higher the ratio, the stronger the bioadhesive bond, indicating better adhesion properties of the tested material.

Figure 4S shows how the microvilli (MV) width and linear density values were measured and calculated using ImageJ software (version ij154). The analysis was done using ImageJ's scale bar measurement tools.

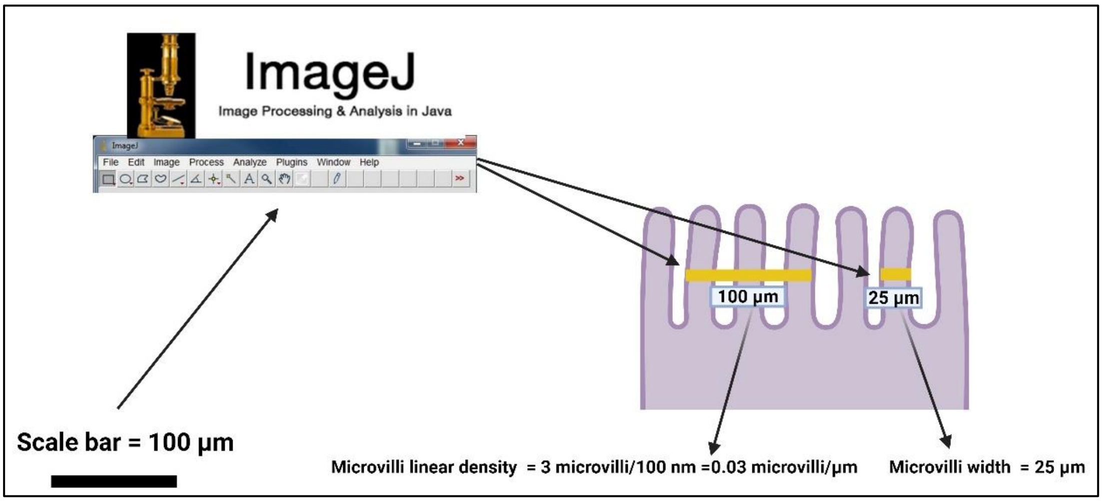

Figure 4S. Schematic depiction of the use of ImageJ for calculating MV width and linear density.

First, the scale bar shown in the selected figure is used to set the pixels-to-length ratio. Next, the width of MV and the length of several (usually 2-4 MVs) MVs are measured. The number of MV per unit of length determines the linear densities.

Figure 5S presents a comparative analysis of MV linear density and width among various species, visually representing the differences that may influence bioadhesion properties in both *in vitro* and *ex vivo* models.

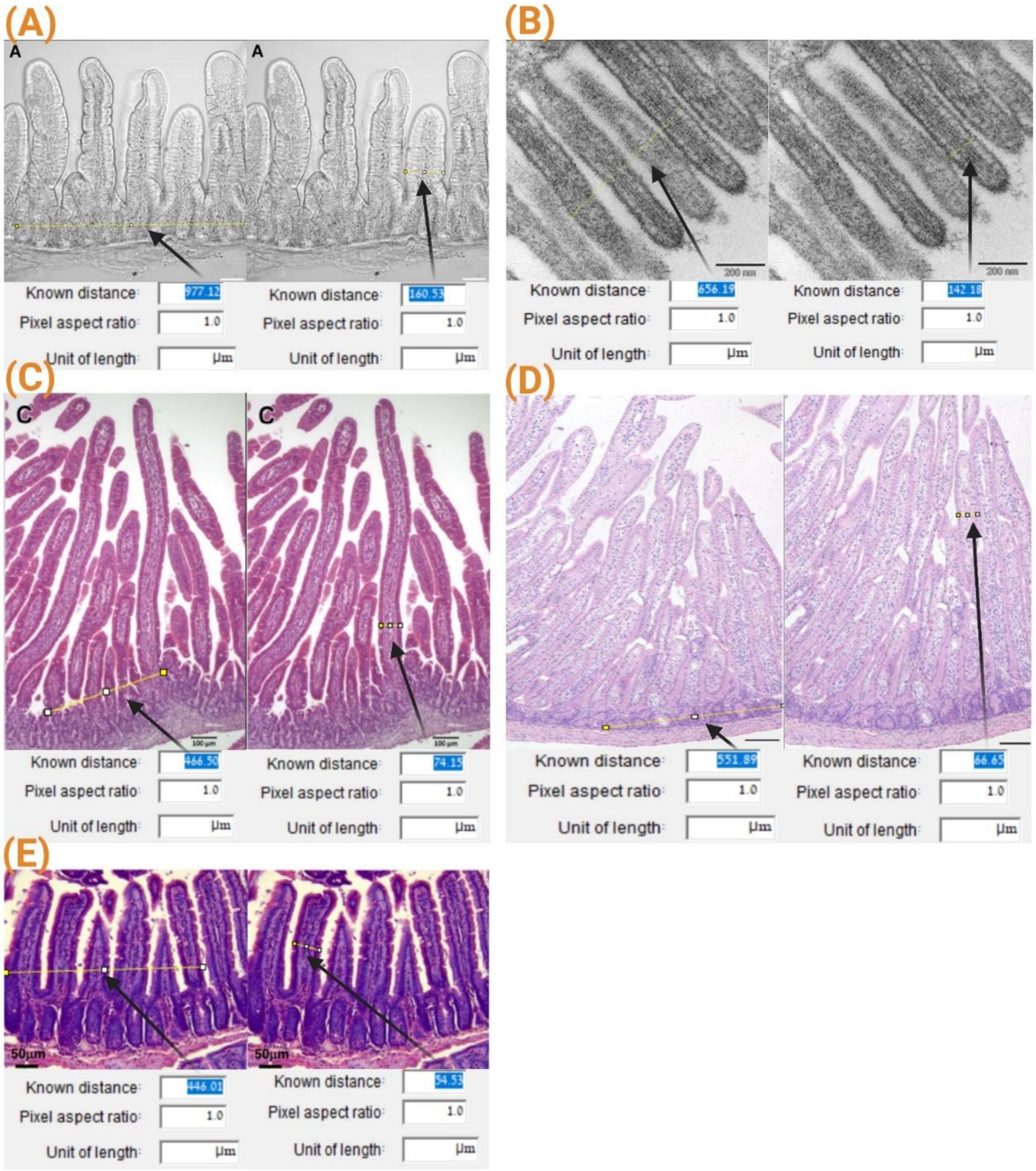

Figure 5S. Comparative analysis of mv density and thickness across species **(A)** Human [1], **(B)** Caco-2 [2], **(C)** Pig [3], **(D)** Sheep [4], **(E)** Mouse [5].

Figure 5S compares MV linear density and width across species, as summarized in **Table 3**. These morphological differences can influence bioadhesion properties, which is crucial for understanding the correlation between MV structure and bioadhesive strength in *ex vivo* and *in vitro* models.

References

1. Liu YA, Chung YC, Shen MY, et al. 678 Whole-Villus Neurohistology With Optical Clearing. *Gastroenterology*. 2014;5(146):S-120.

2. Kucki M, Diener L, Bohmer N, et al. Uptake of label-free graphene oxide by Caco-2 cells is dependent on the cell differentiation status. *Journal of Nanobiotechnology*. 2017;15(1):1-18. doi:10.1186/s12951-017-0280-7

3. Larsson J, Lindberg R, Aspán A, Grandon R, Westergren E, Jacobson M. Neonatal Piglet Diarrhoea Associated with Enteroadherent Enterococcus hirae. *Journal of Comparative Pathology*. 2014;151(2-3):137-147. doi:10.1016/j.jcpa.2014.04.003

4. Flores TJ, Nguyen VB, Widdop RE, et al. Morphology and function of the lamb ileum following preterm birth. *Frontiers in Pediatrics*. 2018;6:8.

5. Maioli TU, de Melo Silva B, Dias MN, et al. Pretreatment with Saccharomyces boulardii does not prevent the experimental mucositis in Swiss mice. *Journal of negative results in biomedicine*. 2014;13:1-8.
